# Application of VR technology in the aircraft cabin design process

**DOI:** 10.1007/s13272-021-00559-x

**Published:** 2021-11-23

**Authors:** Ivana Moerland-Masic, Fabian Reimer, Thomas M. Bock, Frank Meller, Björn Nagel

**Affiliations:** Deutsches Zentrum Für Luft- Und Raumfahrt E.V., Institut Für Systemarchitekturen Der Luftfahrt, Hein-Saß-Weg 22, 21129 Hamburg, Deutschland

**Keywords:** VR technology, Aircraft cabin design, Product design process, User centered design, Human factor in aircraft cabin design

## Abstract

This paper addresses issues currently present in the aircraft cabin design process. It focuses on making the design process more time and cost efficient, while altogether involving the end-users (passengers and cabin crew) in the development process in its earliest stages. By understanding the underlying issues and reasons the cabin is developed according to the current approach, new methods are established and adapted to suit the needs of such a complex process. In this paper, the preposition is made that Virtual Reality is the key technology for achieving the following goals: shortening the initial cabin design process (from sketch to concept design) and including the end-users and their wishes and ideas into the ideation phase. Through cooperation with an external design agency, a Virtual Reality tool is implemented and tested to ensure the theory behind the established design methodology can also be put into practice.

## Introduction

“Dreams are the stuff the future is made of” R. Jensen, 1999.

In the world of aircraft design, most design decisions are based on safety and performance criteria. The aircraft cabin, being an integral part of the aircraft, is no exemption to this rule. The safety of the passengers and crew onboard is the first and foremost requirement for a successful cabin. However, there are plenty other aspects which are perhaps just as important to be considered when designing an aircraft cabin. Comfort of the passengers and their complete in-flight experience is becoming more and more important nowadays. Another factor which cannot be forgotten is the difference between the various cultures where the cabin is being used.

From a different perspective, in the world which is voicing its need for an environmentally friendly way of living, travelling by air has gotten a negative connotation [1]. To counter this phenomenon; air travel has to offer an experience which cannot be met by other means of travelling. As the world is inclined to more micro-travelling and making the journey part of the holiday [2], the challenge to keep air travel as popular as it once used to be, increases.

With all this in mind, the cabin indeed is a rather complex product, with a myriad of different users and stakeholders, each having their own set of preferences and requirements.

When all those aforementioned requirements need to be factored in, the design process tends to become quite lengthy and therefore expensive. This has as direct consequence, that changes in the design in later stages of the process become unwelcome.

To ensure requirements are fulfilled without having to perform radical changes in the mature design of the cabin, it is necessary to include the targeted users already within the conceptual design stages. However, this poses certain challenges. Including potential users into the design process requires understanding the different user groups and their core interests. In addition, the design must be communicated in a way which is understandable for all intended user groups. The former has been widely researched [3, 4]. The latter, however, poses a conundrum. Conceptual design stages currently operate with communication tools consisting of sketches, 3D models and perhaps a simple mock up, the latter often deemed costly and time-inefficient. An example of a potential shortcoming in regular design communication methods is brought up by Ekströmer et al. in their paper on the use of VR in automotive design, specifically lighting design. In their opinion, traditional design methods cannot always bring out the best in all aspects of a designed product when trying to communicating it to all different levels of stakeholders. Especially when lighting is designed, commonplace sketching methods fail short to communicate the experience meant for the user. 3D CAD models are certainly capable to show this aspect of the product, but they have a very long iteration time as these kinds of details are computationally heavy to render [5]. This makes the iterations and changes in these aspects of the design rather undesirable, for it might induce delays in the original planning.

In addition to the aforementioned, stakeholders outside the design team often have difficulties imagining the feeling of the suggested space around them by just looking at a 2D picture. In case of an aircraft cabin, where every inch matters, it is very important to be able to communicate the intended design in a proper way. An emerging technology can provide an answer to this problem. Virtual Reality (VR) has so far been mostly used in the gaming industry, but it is currently finding its way into more professional domains. McLaren Automotive already uses VR to design their next concept cars [6] and Mbryonic has replicated a Boeing 777-300 cabin in a VR environment for marketing purposes [7]. In Hamburg, Airbus has constructed a Cabin Definition Center (CDC), where customers are invited to check if the cabin design is according to their demands. The cabin is shown using highly detailed renderings, and according to the desires of the client, fully detailed and functioning mock ups are built [8].

All the VR applications within cabin design encountered so far are focused on enriching the design process in later stages of design, e.g., the frozen concept, for prototyping, etc. It certainly makes the development process more efficient in comparison to the traditional cabin design process, especially from the clients’ point of view. But, when groundbreaking innovation is required to answer the requests stated above, a more fundamental approach is needed, at the very beginning of the design process.

By incorporating VR technology in the conceptual design phase, the design team could immerse potential users directly into the virtual cabin “construction site”. There they could immediately see the changes the cabin endures when being adapted to their requirements. Using this technology could help the design team not only to widen the design space, but to understand its user even better. This inevitably leads to understanding the users’ needs and complaints and incorporating these at the very origin of the new concept.

This paper will focus on researching the potential of state-of-the-art VR technology and its implications for the aircraft cabin design process. It will explore the possibilities of applying VR in early stages of the design process as well as employment of the technology as an extension tool for the designers themselves. The former will be approached by performing a literature research, establishing the state of the art on the technology. The latter will be based on a use case where a VR environment will be used as a sketching board for 3D drawings of a cabin and its components.

## The traditional cabin design process

**Fig. 1:**
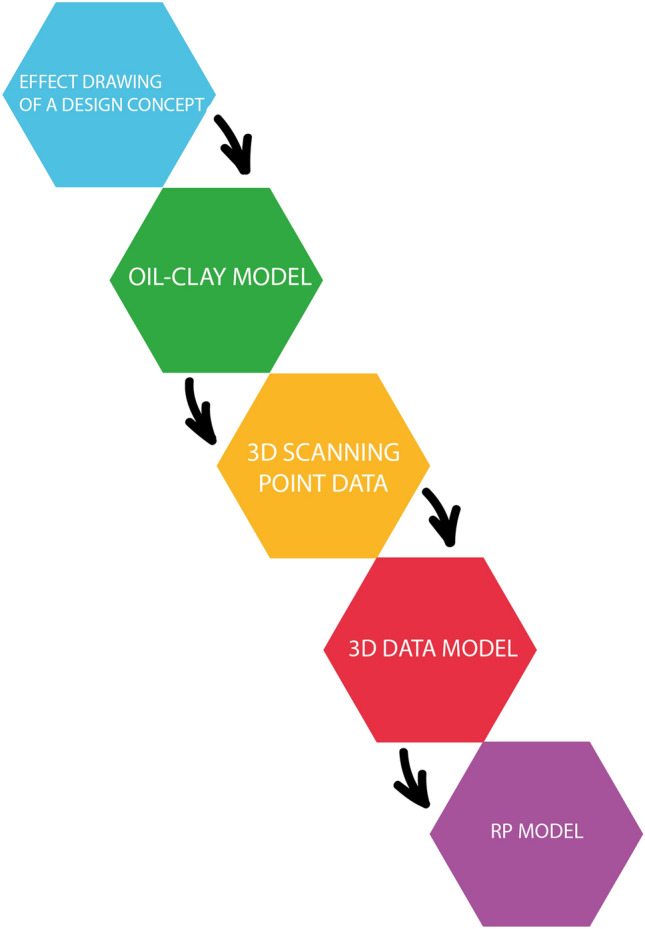
Industrial design process according to Ran and Wang

In their paper on Virtual and Augmented Reality Applications in Industrial Design, Ran and Wang have illustrated a traditional industrial design process [9]. Figure 1 shows their simplified explanation of the process. According to Ran and Wang, the industrial design process roughly follows five steps until the final concept design is reached. This is explained using a motorcycle as an example.

**Fig. 2:**
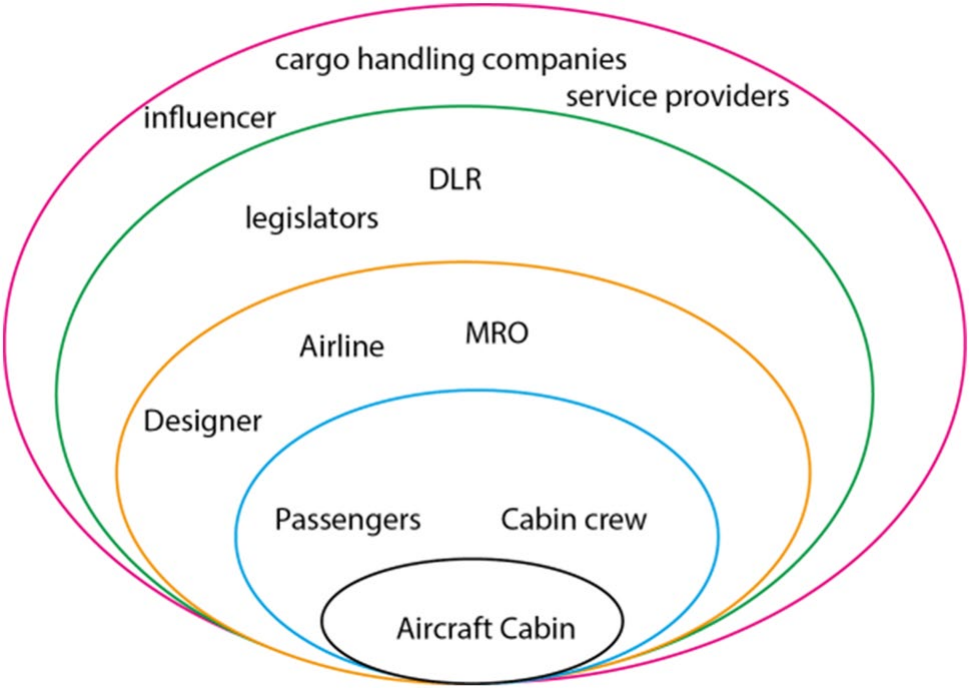
Aircraft cabin stakeholders onion diagram [12]

In case of an aircraft cabin design process, this is a bit more complex. Firstly, in most cases the design team and the “customer” belong to the same company. After a gap (unfulfilled need, shortcoming of an existing product) has been established by a marketing research team, the design team takes over and designs a concept product which can fulfill the corresponding need. The iterations during the design process all take place within the same company, where communication channels are short and, perhaps even more important, all the teams work with the same vision in mind. Secondly, there are not many products available that have to withstand the same level of safety certification as an aircraft cabin. Multiple (non-) governmental institutions are involved in the development process. Some of them only passively, by means of already existing certification legislation [10], others actively, “supporting aviation with global standards for airline safety, security efficiency and sustainability” [11]. In addition, the number of stakeholders is exceptionally high. Figure 2 shows a simplified overview of cabin design case stakeholders.

To get a better understanding of how a cabin design process actually works in practice, an interview was conducted with a highly experienced cabin designer, Thomas-M. Bock, who is nowadays also a member of the Cabin design team at the German Aerospace Center (see Sect. 4.1). Mr. Bock has designed cabin layouts for over 35 years over the span of the entire fleet of Airbus.

**Fig. 3:**
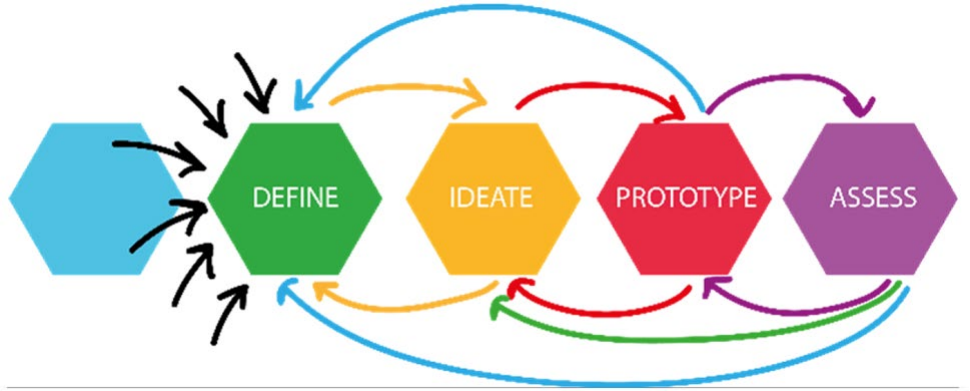
Simplified illustration of the current cabin design process

During the interview, it became clear that the “traditional” approach to cabin design is rather time and cost intensive [12]. For example, while working on a design of a cabin for an America West A319, it took multiple scaled mock ups until the client was satisfied with the outcome. On a different occasion, while designing a cabin for a Thai Airlines A380, more than ten meetings were needed to meet with stakeholders of all the disciplines involved and take down their requirements. These are only a few examples of the elaborate and complex communications necessary for customers to express their desires and the design team to interpret those desires in the right way. The main conclusion of the interview was that in general, a time span of roughly 2 years is what it takes for a cabin to be designed according to the customers’ wishes, including all the necessary adaptations in between. Only then it can be handed over to the companies responsible for manufacturing it. Figure 3 shows that the process begins with a “shot in the dark”, where the design team tries to interpret the wishes of the customers, based on previously held interviews and on their own experience gathered over the course of the years. What follows next is a non-linear process of back-and-forth iterations between customer representatives from different layers of management and different disciplines, the manufacturer and the design team. Once the concept design is frozen (i.e., the design has been approved by all the involved parties), it is handed over to a team (often an external company) to work out the details and produce high resolution renderings and detailed three-views.

## VR in aircraft cabin design state-of-the-art

In 2019, De Crescenzio et al. have conducted a study within Horizon 2020 project CASTLE (Cabin Systems Design Toward Passengers Well-being). In their findings, published in the International Journal on Interactive Design and Manufacturing, they state that VR has been recognized as a powerful tool to get the intended user closer to the product even before it has been manufactured [13]. This study was focused on the evaluation of designed cabins by means of Virtual/Augmented Reality. In this case, VR was used to validate the design approach and strategy in search of innovative cabin design. One of their main conclusions is that VR certainly aids in making the design process more time efficient, less costly and more adaptive to change.

Throughout their research in the scope of the CASTLE project, they apply the so called Human Centered Design Approach (HDC), which aims at making systems usable and useful by focusing on users [14]. Bagassi et al. have discovered that, although this approach has been developed for computer-based interactive systems, it can very well be adapted to any system interface design [15].

In 2019, Ekströmer et al. wrote a paper on VR being a key technology to overcome the shortcomings of conventional design communication methods. Once the experience aspects of a design, such as ambient lightening, spaciousness or 1-on-1 perception of the design, need to be communicated, current tools are either expensive and time consuming (realistic physical mock ups or even prototypes), or fail to communicate the desired effect (2D sketches). 3D models in globally accepted CAD software such as Rhino, Alias or CATIA seem to be able to partway transfer the intended message. These tools are however time consuming, due to the fact that rendering those kinds of effects takes up a lot of computational power and therefore, even with state-of-the-art computers, iteration times remain long [5].

Concept design seems to be only one of the possible application possibilities for VR technology. In 2011, Ran and Wang have proposed to use VR across the entire design process [9]. Even earlier, in 2007, Zeplin has presented an Airbus vision on application of this technology in cabin design [8]. This application, however, is focused on presentation of the fully developed cabin to the customers in the Cabin Definition Center in Hamburg (CDC). Here, customers come to check the design for the last time, choose the colouring or the pattern and discuss the final details. Radical changes in the design are extremely expensive at this point in the design process, and therefore highly undesirable.

To allow for a radical change to take place, the design team has to create a point in the design process where such a change is possible without fundamental change in the planning. Early design stages are the most suitable moment, where everything is still digital and adaption is easy. The downside of changes in this stage is that, as aforementioned, communicating the design to the customer is rather difficult. VR seems to be the pivotal technology to overcome this barrier.

## Application of VR to the design process – the “inhouse approach”

### Team history and goals

Within the Institute of System Architectures in Aeronautics at German Aerospace Center, the main authors of this paper have formed a Cabin design group, tasked with delivering new and innovative cabin concept designs. Currently, the group holds expertise in Product Design, Cabin Design, Context- and User Research and Ergonomics. Central goal of the design group is to enable progress in the field of cabin design by focusing principally on its main users, being the passengers and the crew. Primary driving force of the Design group is the idea that there is a lot to gain if these main stakeholders are involved in design process in its earliest stages. In this particular case, the design group lays an emphasis on the end-users to be the stakeholders whose point of view is crucial to the success of an innovative cabin that can respond to the predicted needs of the future [16]. The vision of the group states that, by satisfying passenger’ needs, perhaps even before they were vocalized; travelling by air could become a positive experience once again. The right in-flight experience offered to the passenger will lead to content customer and perhaps to an increase in the popularity of air travel.

### Design approach

To apply this vision to “every day cabin design work”, a suitable design process needs to be determined. As mentioned before, the cabin is a highly complex product. It is also worth pointing out that cultural differences between the different placement markets are an extra dimension added to the design space. This altogether makes up for a very extensive list of requirements that needs to be considered. To tackle a problem of this involution requires a set of carefully chosen design tools. There is a vast amount of different design tools one can choose from, each tailored to suit a certain spectrum of problems to be solved. For the Ideation Phase, one could think of tools like a Journey Map, Brainstorms Sessions or Co-creation sessions. During the Implementation Phase, a Roadmap, Prototyping and Pilot tests are often used [17].

During their exploratory work on as well cabin designs as the process required for enabling the design, the group has discovered that common product design processes are not directly applicable, due to the complexity of the cabin as a product on many levels. As described by Reimer in his paper on the application of the design thinking method to cabin design, a baseline approach to fragmentize the product to understand it, is required [18]. Design Thinking has proven itself to be a methodology based on a solution-oriented approach to tackle complex problems [19].

**Fig. 4:**
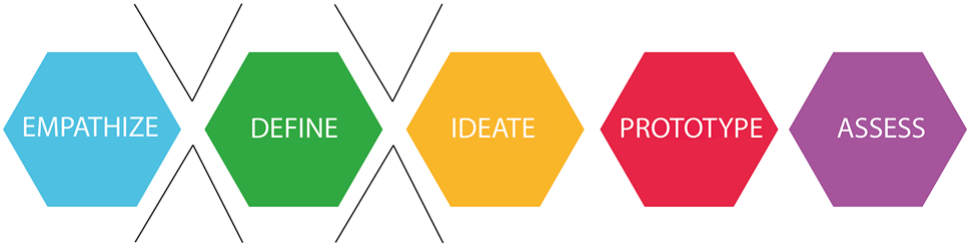
Involvement within the stages Empathize, Define and Ideate is where the highest gain can be made from end-user participation

As stated earlier, another issue that adds to the complexity is that the end users are barely or not at all involved in the process. If there is an involvement of the users in the design process, that happens by the means of questionnaires to validate the new cabin design, or to ask for pain points and issues during the flight with the current cabins [3]. Within several in-depth interviews conducted with potential end users (passengers, crew members and people with reduced mobility), it became clear that this group seeks involvement in the design process of a new cabin. The interviews were conducted online, due to the pandemic restriction, in a one-on-one format. With permission of the interviewee, the interviews were recorded and transcribed, but are according the agreement with the interviewees, not publicly available. In the interviews, the interviewee have clearly vocalized some of the pain points such as a lack of clear overview of the cabin [20] and a lack of space to maneuver a wheelchair in toilets [21]. Incorporating not only their requirements, but moreover their experiences into the design process in its earliest stages, thereby making the end-users pseudo-members of the design team, could provide valuable insights. In Fig. 4, points in the design process where the highest benefit could be made from the user involvement are identified.

**Fig. 5:**
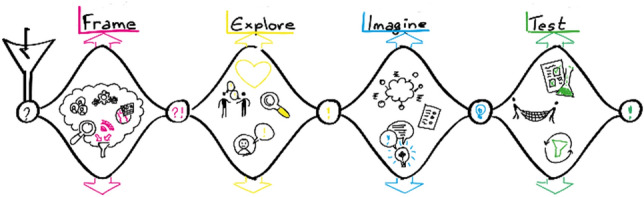
Co-design process (Adapted from Auckland designlab)

This holds true for the design of the everyday products, where for example, co-design is used as a very efficient design process [22]. As Sanders and Stappers described in one of their recent papers, co-design is defined as collective creativity applied over the whole design process, from the very early stages until the end [23]. This means that users can influence the development of the product throughout all design stages, being concept design or detailed design (Fig. 5).

Recent studies show that Human Centered Design can also be applied to cabin design to make it more applicable to the needs of the users [15]. However, whereas HCD ensures the designs are made *for* the user, co-design enables designing *with* the user. In short, co-design, also known as participatory design, has empowering central, meaning that the ones affected the most by the design, should be given a chance to change and adapt the design to their true needs [24].

To be able to include the users in the design process, a communication channel needs to be established to understand the users on one hand and communicate the interpretations of that understanding back to the users on the other. Virtual Reality is a potential tool which could compensate for the shortcomings of the common design tools and provide an answer to this challenge. The ability to let the users immerse in the concept they are helping to be created, allows them to directly see the consequences of their requests. Engaging the users as well as other stakeholders in this way and at the very beginning of the design process, could make a big difference in the time span of cabin concept design.

### Applying VR to the cabin design process

**Fig. 6:**
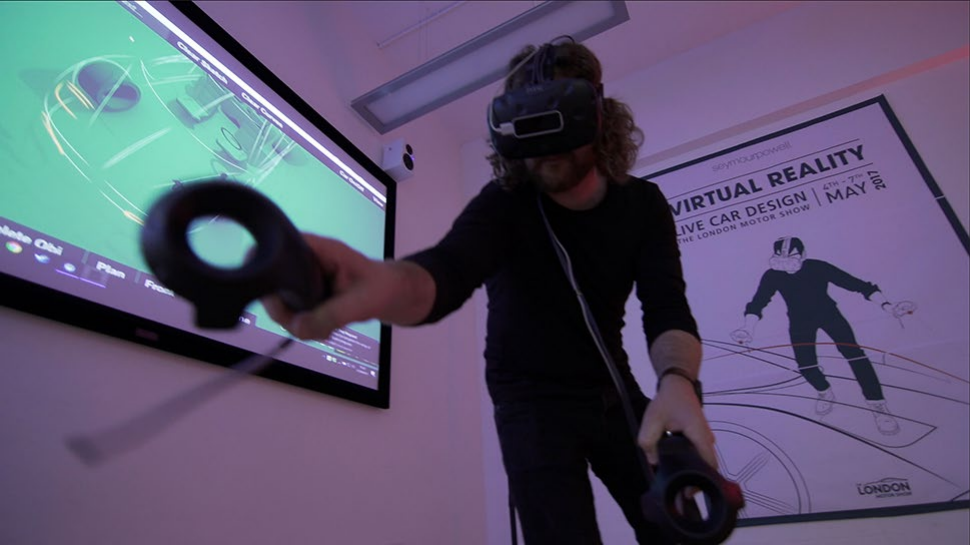
A car is being designed on a 1-to-1 scale with RealityWorks (courtesy of SeymourPowell)

As mentioned in the prior sections of this paper, VR seems to provide the answer to the issues that arise during a cabin design process. To apply VR to the design process, a tool is needed which is intuitive to use without overwhelming the user or expecting any kind of designer background on the one hand and providing a communication platform between different levels of stakeholders on the other hand. Reality Works, created by SeymourPowell, seems to offer that exact balance (see Fig. 6).

**Fig. 7:**
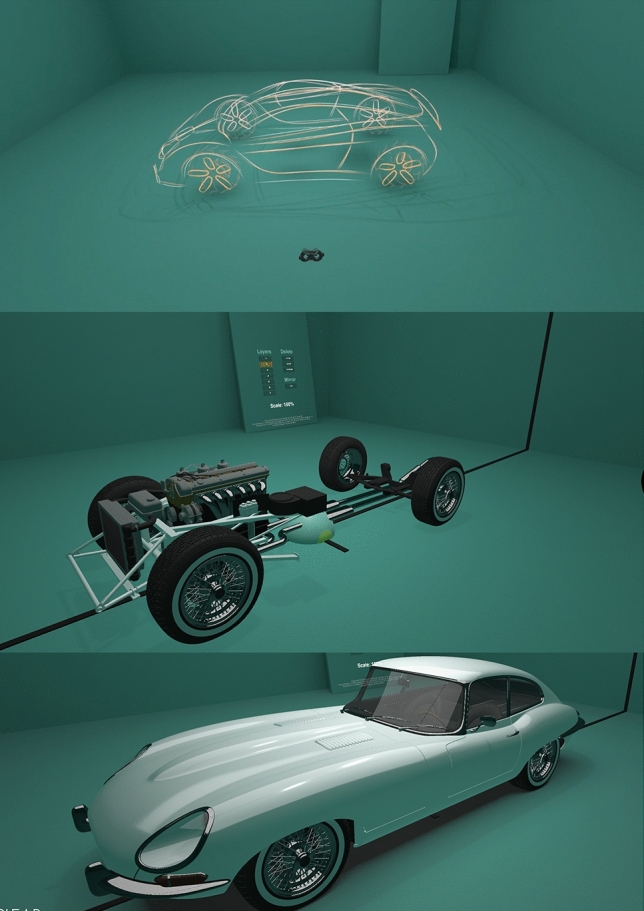
Three different stages of detail possible in RealityWorks (courtesy of SeymourPowell)

Based in London, SeymourPowell’s multidisciplinary team of designers, engineers, and technologists specialise in creating world-first product and brand experiences that define the future. As an answer to the global progress, they developed Reality works in 2017 as the world’s first VR design and collaboration tool developed specifically for transport design; built to enable a more immersive, empathetic, and streamlined design process that could harmonise the agendas of designers, engineers, and regulators in a single dynamic process. Revealed to the world at Tedx and SXSW in 2018–2019, the tool is currently being used across the globe to create and review designs at full scale and in the contextual environments they are meant for; slashing the time and cost it takes to go from early moments of inspiration to concept visualisation and validation (Fig. 7).

In response to the shifts that have taken place across the globe due to the Covid-19 pandemic in 2020, the tool has rapidly been adopted and tailored with new toolsets to accompany the core functionality for various new use cases; enabling new methods of working between non-local teams where close proximity is not currently an option [25].

Over the past year, working closely with the designers from the SeymourPowell, a set of requirements has been established that RealityWorks has to fulfill to suit the needs of the future cabin design process. Once adapted to the needs of the design team, this tool will form the basis for future design work in VR.

#### Use case

In the beginning of 2020, a pioneering project started, called InDiCaD (Innovative Digital Cabin Design). In cooperation with nine partners from all over the German Aerospace Center, each with a different expertise, the cabin was to be designed and thereafter incorporated into a large workflow where among else the structural calculations can be made. Projects’ main goal is summarized in its name: to design innovative aircraft cabins in a completely digital surrounding. It should follow the complete design process until the prototyping phase. The Design group has been tasked with full user and context research, trend and stakeholder analysis as well as delivering the preliminary and final concepts of the cabin.

Considering the fact that this has not been done before using a team consisting only of inhouse engineers and scientists at DLR, this project seems to be a very fertile ground for a test case in applying VR in the cabin design process. At the moment of writing this paper, the project team has finished the research and definition phase of the project and the ideation phase is about to commence. There have already been numerous brainstorming sessions, where the project team has come up with different possibilities for an innovative aircraft cabin which are very well worth of researching. One of those ideas will serve here as an example for application of VR.

##### Pilot use case

To determine whether the RealityWorks is applicable in this particular design problem, a pilot use case has been created and executed. The test subjects were members of the design team themselves. With a group of three (the author included), they hold different levels of experience in cabin design; whereas the colleague doing the 3D model sketches has over 35 years of experience, the other two members hold around 5 years of design experience. In the Table 1, an overview can be found of the used software and hardware for the pilot use case. These tools will also be used for the continuation of the project.

**Table 1:** Software and Hardware used in the Pilot

Type of model	Used software	Used hardware
2D sketch	Adobe Photoshop	WACOM Cintiq Pro 24 DELL Workstation: Intel(R) Xeon(R) Silver 4110 CPU @ 2.10 GHz 2.10 GHz 128 GB RAM 2 × NVIDIA Quadro RTX 6000
3D model	Rhino 3D	DELL Workstation: Intel(R) Xeon(R) Silver 4110 CPU @ 2.10 GHz 2.10 GHz 128 GB RAM 2 × NVIDIA Quadro RTX 6000
VR sketch	RealityWorks	HTC VIVE Pro Eye Wireless DELL Workstation: Intel(R) Xeon(R) Silver 4110 CPU @ 2.10 GHz 2.10 GHz 128 GB RAM 2 × NVIDIA Quadro RTX 6000

**Fig. 8:**
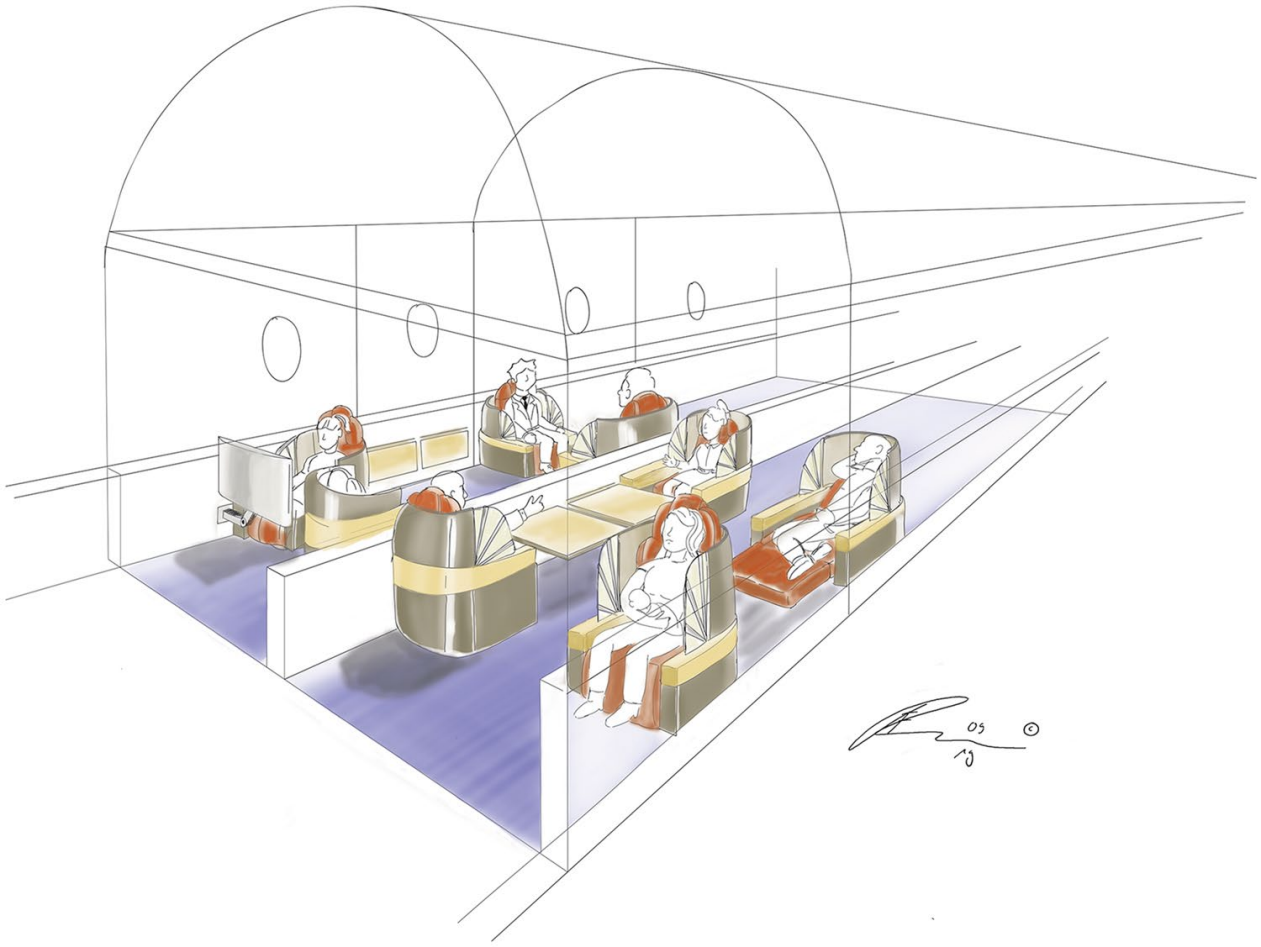
2D sketch of a business class arrangement, effort: 2 h (designer: F. Reimer)

Firstly, a 2D sketch of a business class arrangement has been drawn, presented in Fig. 8. It is already clear from the first glance that the level of detail transferred using such a sketch is limited. The sketch speaks to the viewer as being intriguing, but one cannot take a look behind the chairs or change the coloring of the elements without extensive effort.

**Fig. 9:**
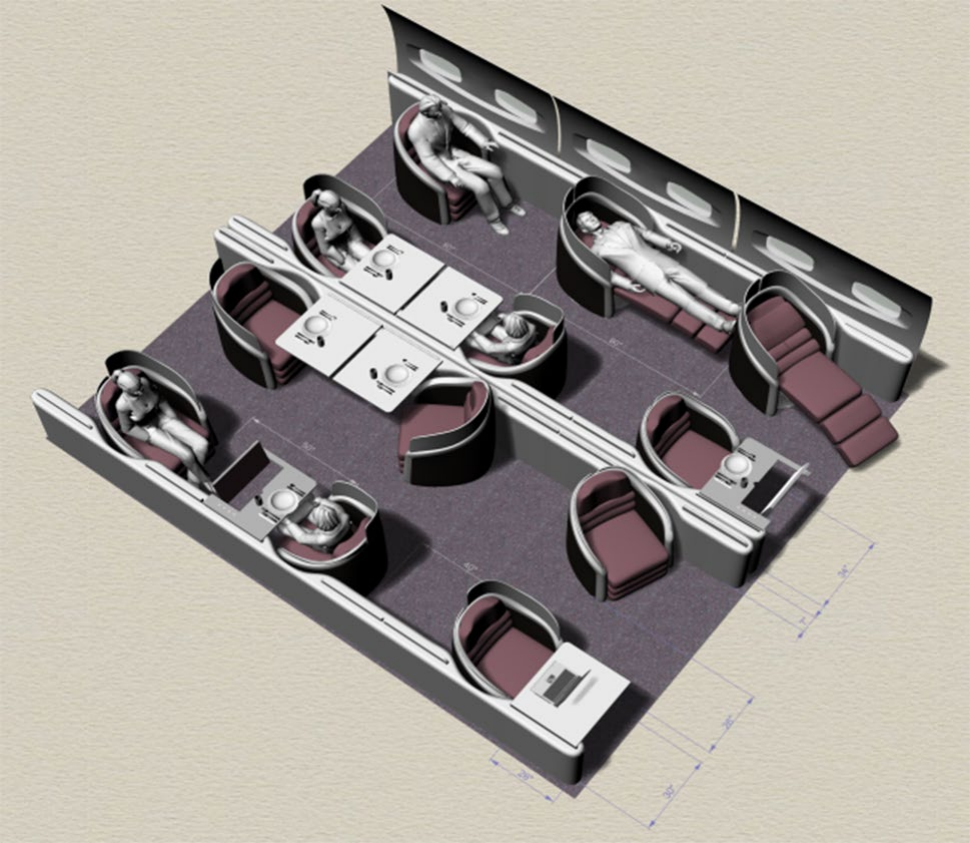
Animated 3D model of the business class arrangement, effort: 3 + weeks (designer: T.-M. Bock)

Based on the 2D sketch in Fig. 8, a 3D model is made in Rhino, shown in Fig. 9. The level of detail is already much higher; the model can be pivoted and colours can be changed. There are even measurements laid out on the floor so that the viewer can get a feeling of size of the model. However, changing the textures, materials and lighting, although possible, is still time inefficient, as the renderings need to be created again. And even though the viewer can take a look behind and under the chairs, it still remains on the computer screen in front of the viewer, scaled to fit the screen. It is still very difficult to get a feeling of the real size of the cabin as it is to get an optical impression of the colours and the textures.

**Fig. 10:**
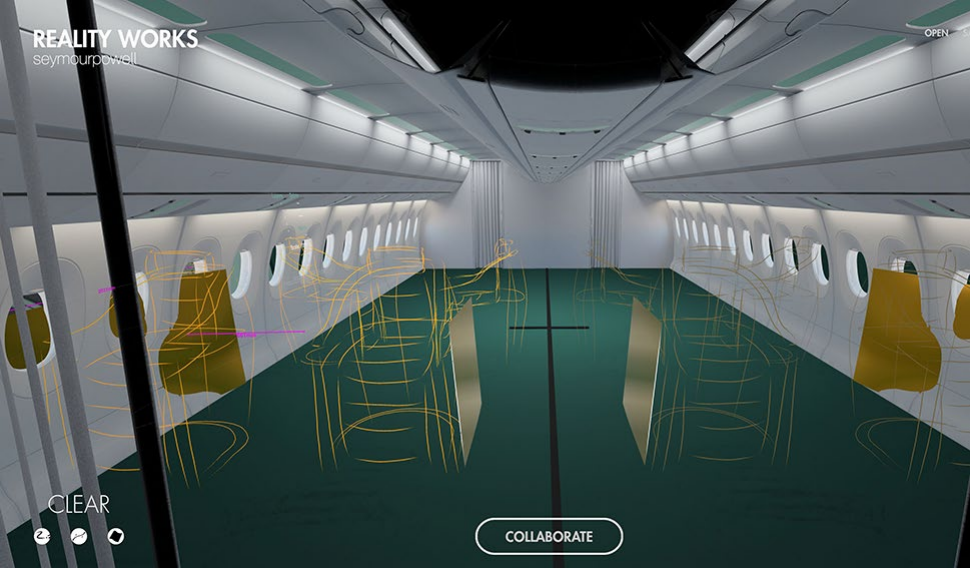
VR model of a business class, quick sketch (designer: F. Reimer)

Figure 10 shows a sketch of the same business class in RealityWorks. The first thing that cathes the viewers eye, is that the designer is sketching directly in an aircraft cabin. The tool allows for uploading own 3D models, made by a surface based modelling software (in this case Rhino). This enables the designers to use already existing designs as underlays, for the purpose of inspiration or even design corrections of the already existing design.

To elaborate, the customer could express a desire to change something in the 3D model. By uploading the 3D model in RealityWorks and “walking through” it wearing VR equipment, they can communicate their desires directly to the designer walking along side of them using the same equipment. Changes can be directly applied to the model and approved by the customer without any delays.

**Fig. 11:**
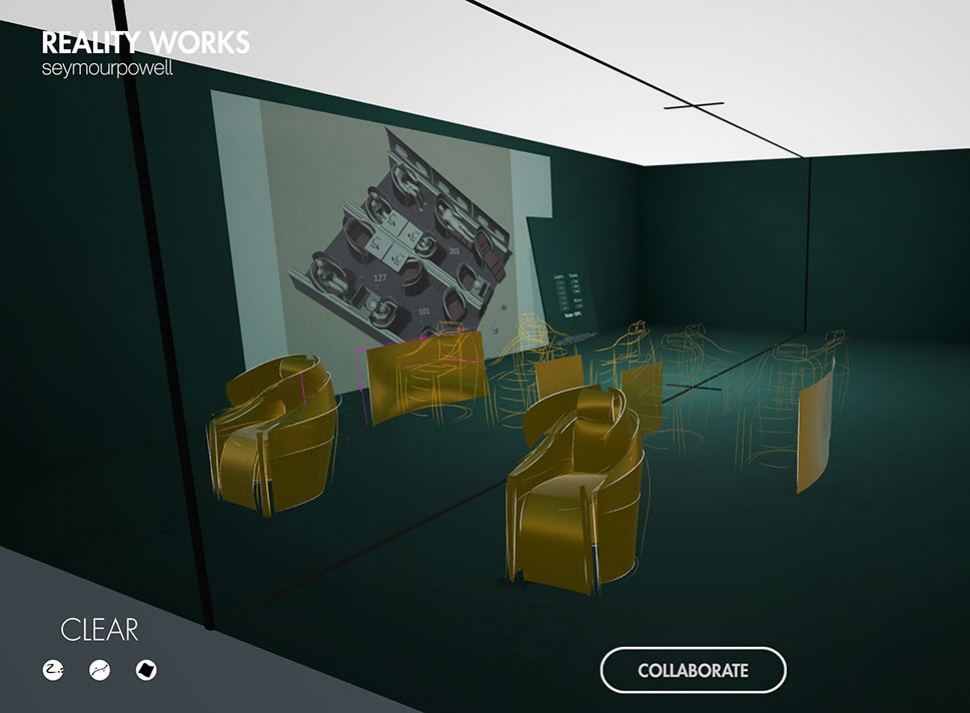
Business class with 3D model in the background, quick surface modelling effort: 20 min (designer: F. Reimer)

Figure 11 shows a more elaborate sketch of the business class arrangement, where the seats are overlaid with surface to offer more content to the sketch. The customer/user can walk around the seats; look at them from all sides. By combining VR with AR by installing trackers on the chairs properly placed in the real space, it is even possible for the customer/user to sit on the chairs and experience the cabin from that point of view. This way, the optical impression can be tested.

**Fig. 12:**
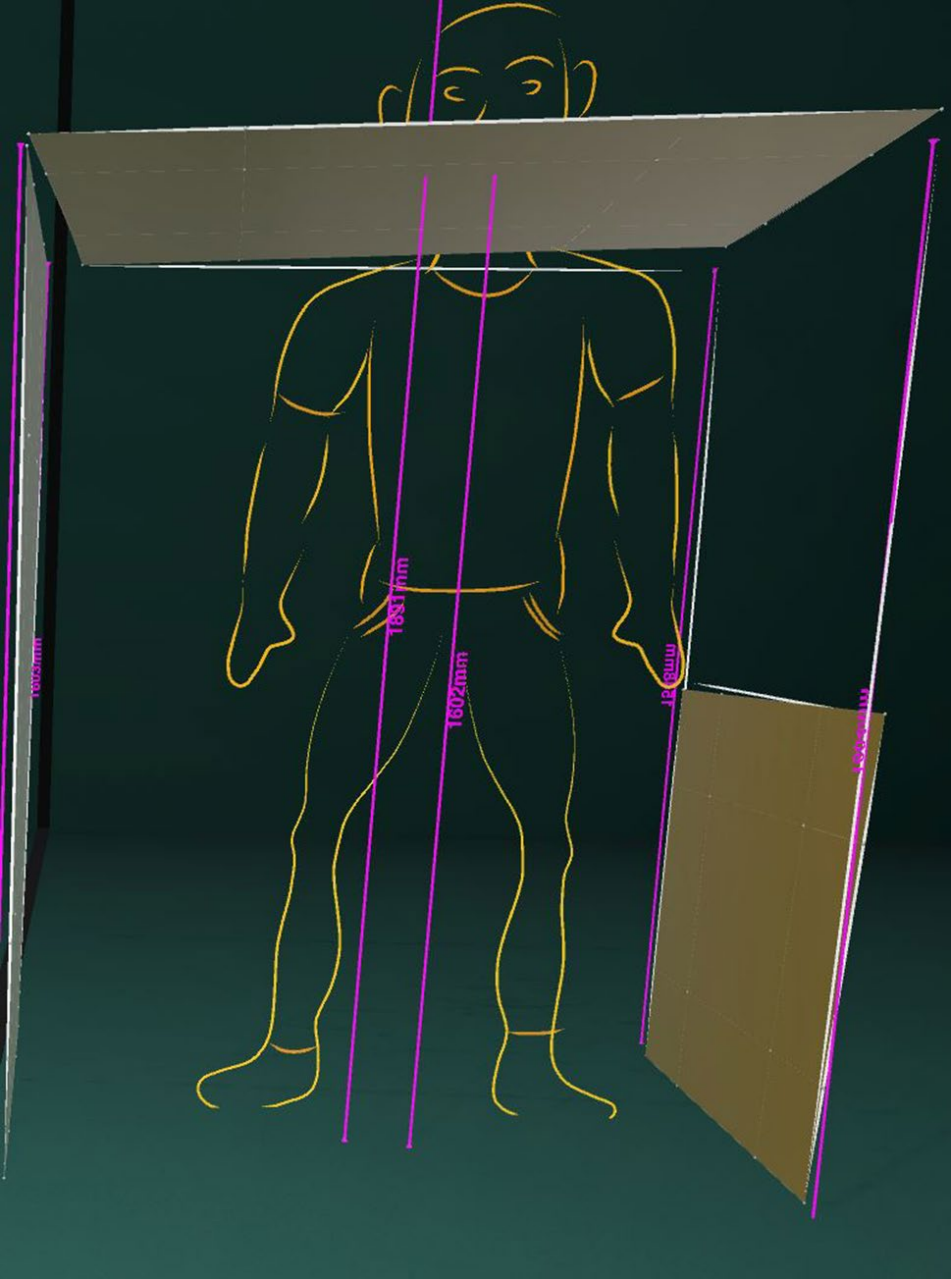
Sketch of a human figure (percentile 90) in a lower deck (1.60 m height) (designer: F. Reimer)

To illustrate the spaciousness effect of the VR tool, Fig. 12 shows a 90-percentile human figure standing up straight in the lower deck of an A350, which is only 1.60 m high. When designing a cabin for the lower deck, for example, this insight is very important to have already at the beginning, because it forms a massive constraint on the design space available. Even though it is common knowledge for a cabin designer, experiencing it themselves provides an extra dimension and a better understanding of this issue.

All of the sketches were captured during simulations in VR, where one of the designers was sketching and the other was monitoring the process on the external screen.

##### Future use case(s)

The pilot use case was well received by the design team and its promising results have ensured the continued use of the VR tool. One of the features of the tool that astonished the team was the intuitivism with which it could be used. The learning curve was very steep and within minutes the designer was able to use it. No matter how promising the initial results are, the tool still needs to be properly evaluated. This will take place in the next phases of the project.

As the InDiCaD project progresses, the intention is to introduce the tool to the rest of the project team and first potential users and to apply it during the Ideate and Prototype phase (see Fig. 8). This would be one of the first cases where people outside of the design team are involved in the design of the cabin starting from the first sketches. The idea is to let the project team take an active part in designing, meaning literally taking the controller and sketching their own ideas in the open space. This part of the project is planned for the following project year.

The validation phase of the project will also take place in VR, using the same tool. This has as an advantage that the project team is already familiar with the tool and that it does not distract them from the design they are working on.

#### Benefits of VR in cabin design

At the end of the pilot use case, it appeared that VR as a technology and RealityWorks as a tool implementing that technology have even more benefits than only communicating the designs with the customers. The technology indeed moves the bottlenecks to the early concept design stages, where changes are still easily applied. Designers can easily discuss modifications with users/customers, and directly adapt the model to show the consequences of the desired alterations. In addition to this, it is a very powerful tool for quick sketches while brainstorming. Up to four people can actively make changes to the model, where a multitude of invitees can passively observe the model and participate in the discussion. The latter actually has an extra dimension suiting the needs of the team under the current circumstances. As the world is battling the Covid-19 exposure and most of the colleagues are in Home Office, working together becomes something of a challenge. Especially for designers, where new concepts need to be discussed and one needs to be able to show what (s)he means by the expressed remarks, it is very important to be able to interact with one another. VR technology allows for this remote interaction by means of collaborating in the same scene in the Reality Works.

#### Risks, constraints and uncharted territory

Although the benefits of VR technology as well as of the RealityWorks tool are convincing, there are also disadvantages which need to be considered. Firstly, to use VR, special equipment is needed. High performance computers with strong graphic cards are necessary, as well as VR headsets and controllers. Each member of the team needs his/her own set, to enable simultaneously working on the same model. This requires a certain investment. Secondly, the technology is still rather new and not widely used. Wearing the headset and using the controllers require getting used on, which might imply a certain level of distraction to the user. How big this distraction is, depends on the person and his/her previous experience with VR. This however should ebb once the user is more proficient in dealing with the equipment. To counter this, a pilot run is necessary, where the test persons are allowed to use the VR equipment unrelated to project work, to get used on the equipment. Thirdly, another issue that arises when using VR is currently under research in the German Aerospace Centers’ Institute of Aerospace Medicine, where the hypothesis of VR having health implications (such as getting nauseated during extensive use) is tested. This research and its results will be watched closely for any indications this might have for the application of VR proposed in this paper. Finally, personal reactions to wearing the headset should not be treated lightly. Once the headset is on, the person using it cannot see anything in the outside world, but is very aware of the fact that other people around him/her still can see him/her. This can be experienced as very uncomfortable and therefore influence the perception and experience of the intended design. This can be countered in two ways. The test person could get the time needed to adjust to the idea of being exposed and blinded to the real world. This however, might take a substantial amount of time, which is contradictory to the intended time saving effect of the technology. Another way to deal with this issue is to separate the test person from the spectators by the means of a physical enclosure (e.g., using a separated VR space).

## Summary and outlook

This paper describes the application of VR technology in the cabin design process in the earliest stages of the design. It focuses on making the design process more efficient by means of enriching the communication between different stakeholders and stakeholder levels with immersive, real size imagery of different detailing level. In contrast to traditional design approaches, advanced VR technologies enable experiencing the design full scale, without notable time and cost investment. By involving the stakeholders, particularly end-users, in the Inspiration and Ideation Phase, a lot of time can be gained and a lot of effort can be saved when compared to stakeholders’ involvement in later stages of the design process.

In the upcoming couple of months, new projects are about to start that require innovative cabin designs. One of these is for example HorizonUAM, a project about urban air mobility, where the cabin design group is tasked with the research and design of a suitable cabin. Starting early next year, project CHASER will be launched, dealing with a future generation Search and Rescue helicopter, where the group will deliver a cabin for doctor and patients according to the list of requirements given by the German Aerospace Centers’ Institute for Aerospace Medicine. The InDiCaD project will continue to progress for the upcoming 3 years, where the cabin of the future will get its final shape.

All these projects are fertile grounds for application of VR technology. The future work of the authors will be focused not only on applying the technology to gain results, but also to research the consequences this technology has on the design process and its designers.

## Data Availability

Available per request to main author.
